# Publisher Correction: Chip-scale atomic diffractive optical elements

**DOI:** 10.1038/s41467-019-11529-7

**Published:** 2019-08-12

**Authors:** Liron Stern, Douglas G. Bopp, Susan A. Schima, Vincent N. Maurice, John E. Kitching

**Affiliations:** 1 National Institute of Standards and Technology, Time & Frequency Division, 325 Broadway, Boulder, CO 80305 USA; 2 Department of Physics, University of Colorado, Boulder, CO 80309 USA

**Keywords:** Atom optics, Micro-optics, Atomic and molecular interactions with photons

Correction to: *Nature Communications* 10.1038/s41467-019-11145-5, published online17 July 2019.

The original version of this Article contained an error in the spelling of the author Liron Stern, which was incorrectly given as Stern Liron. This has now been corrected in both the PDF and HTML versions of the Article.

