# Supplementary material for: Chip-scale atomic diffractive optical elements
Source: Nat Commun. 2019 Jul 17;10:3156. doi: 10.1038/s41467-019-11145-5 (PMC6637105; doi:10.1038/s41467-019-11145-5)

**Chip-scale atomic diffractive optical elements – Supplementary**


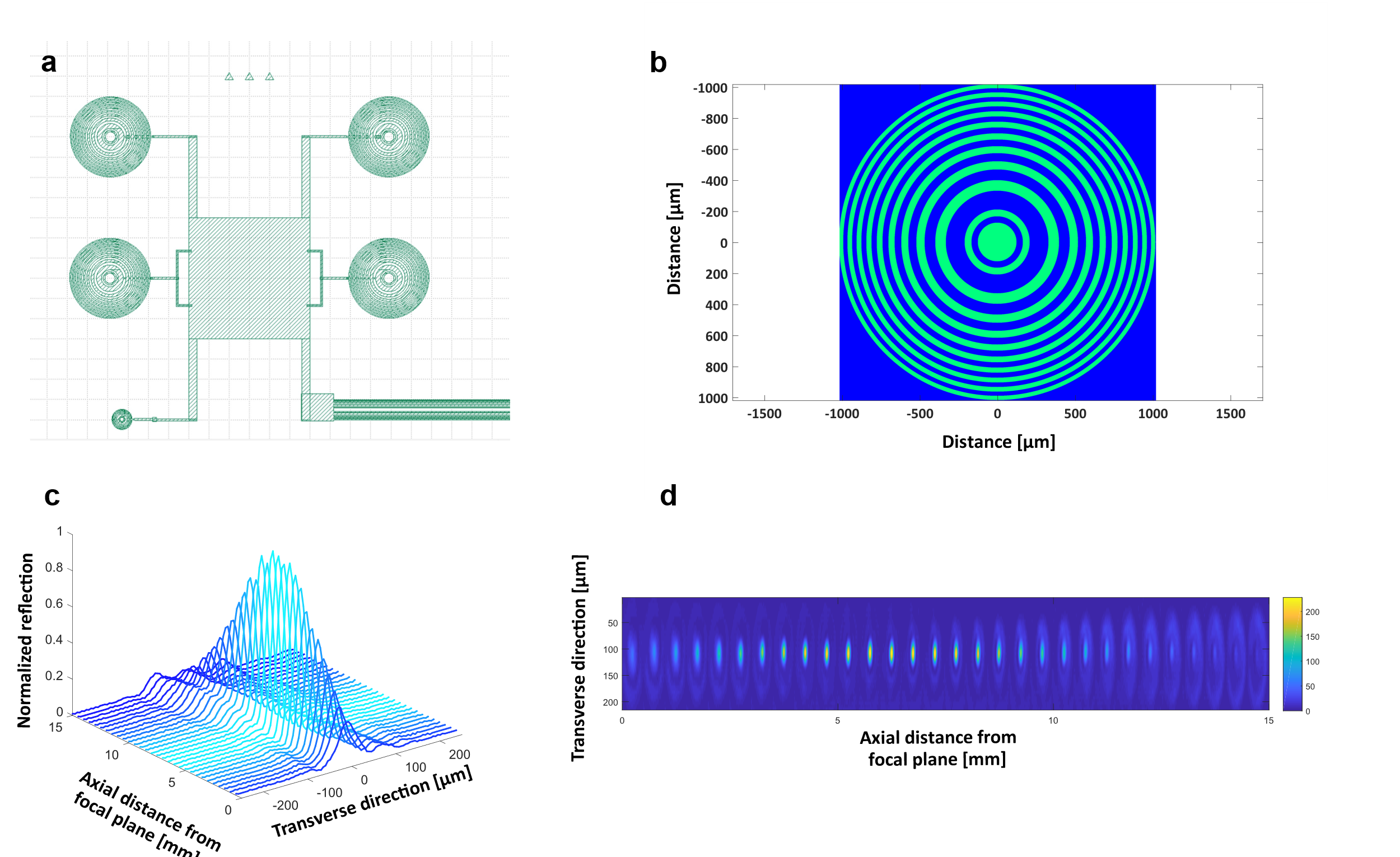


Supplementary Figure 1 | Layout and non-atomic characterization of Fresnel lenses a) schematic layout of the device presented in figure 1c of the main manuscript. The dimensions of each cube in the layout is 500 µm b) typical dimensions and layout of the Fresnel lens b) normalized reflection profile as function of axial distance and one of the transverse directions of the Fresnel lens without active atoms d) Stacked images of the Fresnel lens across the focal point

**
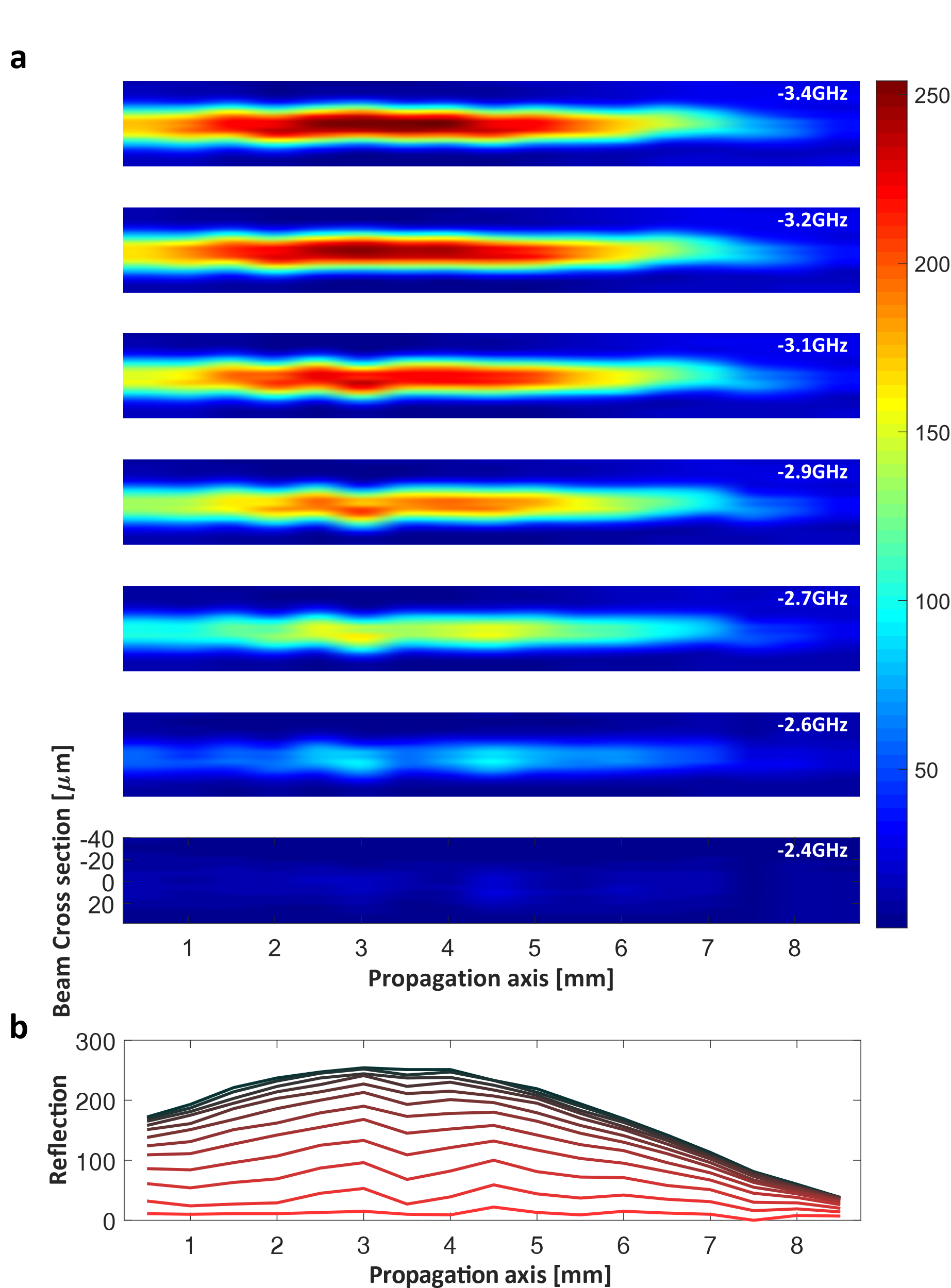
**

Supplementary Figure 2 | Fresnel atomic-lens detailed spectral characterization a) Beam cross section as function of propagating axis, for different detuning (across 1GHz total detuning) b) The gradual transition of the lens operation around the center of axis for different frequency detuning’s (corresponding to different phase responses of the atomic medium).

Supplementary Movie 1

Fresnel lens atomic diffractive element: evolution of focal plane intensity with detuning


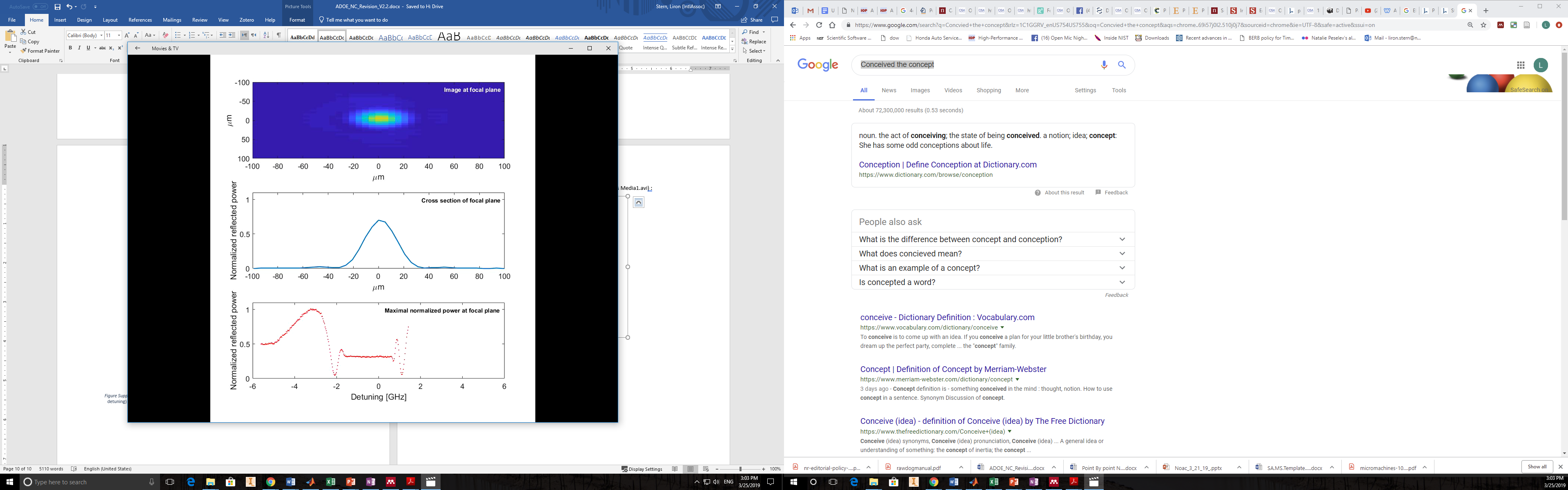

Supplement: Supplementary file 1 — Supplementary Info [file 41467_2019_11145_MOESM1_ESM.docx]
